# Learning Linear Spatial-Numeric Associations Improves Accuracy of Memory for Numbers

**DOI:** 10.3389/fpsyg.2016.00024

**Published:** 2016-01-21

**Authors:** Clarissa A. Thompson, John E. Opfer

**Affiliations:** ^1^Department of Psychological Sciences, Kent State University, KentOH, USA; ^2^Department of Psychology, The Ohio State University, ColumbusOH, USA

**Keywords:** numerical estimation, memory, linear representation, logarithmic representation, development

## Abstract

Memory for numbers improves with age and experience. One potential source of improvement is a logarithmic-to-linear shift in children’s representations of magnitude. To test this, Kindergartners and second graders estimated the location of numbers on number lines and recalled numbers presented in vignettes (Study 1). Accuracy at number-line estimation predicted memory accuracy on a numerical recall task after controlling for the effect of age and ability to approximately order magnitudes (mapper status). To test more directly whether linear numeric magnitude representations caused improvements in memory, half of children were given feedback on their number-line estimates (Study 2). As expected, learning linear representations was again linked to memory for numerical information even after controlling for age and mapper status. These results suggest that linear representations of numerical magnitude may be a causal factor in development of numeric recall accuracy.

## Introduction

Remembering numeric information is an important part of modern life. Sometimes numbers must be recalled verbatim (e.g., personal identification, phone, and flight numbers); other times remembering the gist of numeric information will suffice (e.g., savings account balances, temperatures, number of students in a lecture hall). Children’s ability to recall exact and gist numeric information improves greatly with age and experience (Dempster, 1981; Brainerd and Gordon, 1994).

To explain age-related improvements in numeric memory, early research pointed to cognitive changes that applied equally to memory for numbers and other types of information (e.g., letters, syllables, animal names). Among these 10 potential causes were improved use of strategies—e.g., rehearsal (Ornstein et al., 1975), grouping (Easby-Grave, 1924; Estes, 1974), chunking (Simon, 1974; Chi, 1978), and retrieval selectivity (Samuel, 1978)—and non-strategic variables, such as speed of item identification and item ordering (Chi, 1977), attentional capacity (Dempster, 1978), resistance to interference (Leslie, 1975), search rate (Keating and Bobbit, 1978), and output buffer (Baddeley et al., 1975). However Dempster (1981) found that age differences in speed of number identification was the only variable that reliably accounted for age differences in children’s memory for numbers.

**FIGURE 1:**
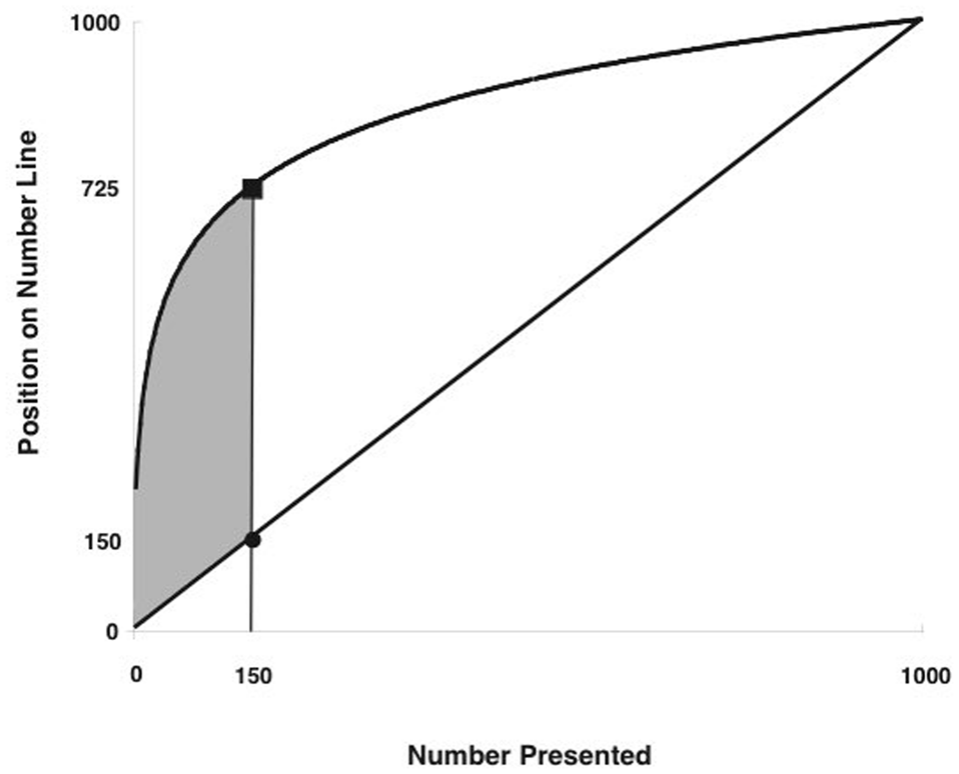
**Logarithmic and linear functions.** Distance between representations is greatest at 150 (725 vs. 150); this means that the logarithmic function increases more than the linear representation between each successive pair of numbers up to 150, but increases less than the linear function above 150. Thus, numbers below 150 are more discriminable in the logarithmic representation, and numbers above 150 are more discriminable in the linear representation.

Modern research on memory development and numerical cognition, however, points to another potential cause for age-related improvements in memory for numbers, namely developing representations of numerical magnitudes (Brainerd and Gordon, 1994; Thompson and Siegler, 2010).The basic premise of this account is that the distinctiveness of a trace in memory determines in large part how easily it can be retrieved, with information leaving the most “fuzzy trace” in memory being the most difficult to recall (Greene and Crowder, 1984; Brainerd and Reyna, 1990). The generalization of this account has an interesting implication for children’s memory for numbers: because children initially represent large numeric magnitudes as being less distinct than small numeric magnitudes (as on a logarithmic ruler; see Siegler et al., 2009; **Figure 1**), large numbers are also more difficult for young children to retrieve from memory than smaller numbers (Thompson and Siegler, 2010). Additionally, within any age group, children with the least accurate memory for numbers are also the ones most likely to estimate the positions of numbers on number lines to increase logarithmically (rather than linearly) with actual value (Thompson and Siegler, 2010).

If true, this account has important theoretical and practical implications. Theoretically, it might explain the previously observed association between age and ability to remember numbers (e.g., Brainerd and Gordon, 1994). Practically, it suggests that children’s memory for numbers could also be improved by engendering the logarithmic-to-linear shift observed in previous training studies (e.g., Opfer and Siegler, 2007; Opfer and Thompson, 2008; Thompson and Opfer, 2008). Testing this practical implication is also theoretically interesting because it could provide evidence for a *causal* link between numerical representations and memory, as opposed to just a correlation that might be equally well-explained by associations with a third variable (e.g., processing speed, working memory, proportional judgment skills).

We had hypothesized that the link between numerical representations and memory accuracy was causal largely due to theoretical problems in domain-general accounts of number-line estimation development. The major problem for the domain-general account is that number-line estimation ability does not develop like a domain-general ability. For example, when the same child is given two different number-line tasks (e.g., 0–100 and 0–1000), she typically provides a linear series of estimates for the smaller scale and a logarithmic series of estimates for the larger scale (Siegler and Opfer, 2003). Given that the same child cannot have both a short and long working memory span at the same time, therefore, a general cognitive skill like working memory cannot explain these two different patterns of estimates. In addition, evidence that has been adduced in support of one domain-general ability, proportional reasoning skills (Barth and Paladino, 2011; Slusser et al., 2013), appears to be an artifact of a very specific, atypical procedure for introducing number-line problems (Opfer et al., 2016). Thus, we were skeptical that the previously observed correlation between number-line estimation and memory accuracy was merely due to the development of some general cognitive skill.

### The Current Study

The current studies were designed to test for a potential causal link between children’s numerical representations and their numerical memory. In Study 1, Kindergartners, second graders, and adults estimated numbers in the 0–1,000 range and recalled numbers presented in meaningful vignettes. The purpose of Study 1 was to investigate the unique contributions of both age and quality of numerical representations to memory accuracy in numerical recall, as well as to identify children who would benefit from training in Study 2. It was an open question as to whether Kindergartners would be able to learn to produce a linear series of estimates in the 0–1,000 range after receiving feedback. We tested all participants in the 0–1,000 range because we wanted to assess developmental improvements in the relation between estimation and memory for numbers across the lifespan. Also, this was the numerical range that was used in previous research (Thompson and Siegler, 2010).

In Study 2, Kindergartners and second graders received training on the number-line estimation task, following the procedure used in Opfer and Siegler (2007). Our goal in Study 2 was to investigate whether adoption of linear spatial-numeric associations on the number line estimation task would improve recall of numerical information. We were particularly interested in memory for large numbers (>150) because they were much larger than those for which children received training (150), yet were predicted to elicit the greatest improvements by the logarithmic-to-linear shift account.

## Study 1: Age Differences in Numerical Estimation and Memory Accuracy in Numerical Recall

### Method

#### Participants

Participants were 14 Kindergartners (*Mean age* = 6.25 years, *SD* = 0.39 years; 50% girls; 93% Caucasian, 7% Asian), 63 second graders (*Mean age* = 8.31 years, *SD* = 0.33 years; 45% girls; 95% Caucasian, 3.1% Asian, 1.6% Biracial), and 28 adults (*Mean age* = 20.07 years, *SD* = 2.3 years; 50% women; 71% Caucasian, 29% Asian). Children were recruited from three schools in southwestern Pennsylvania. On average, 11% of all children attending these schools were eligible for free or reduced-cost lunches; the Pennsylvania state average is 33%. Adults attended a private university in southwestern Pennsylvania and received partial course credit for participating. This study was carried out in accordance with the recommendations of the Carnegie Mellon University IRB with written informed consent from the parents/guardians of all subjects and verbal assent from all child participants.

#### Tasks

##### Numerical estimation

Participants were asked to estimate the position of 22 sequentially presented numbers on a line, where the left end was labeled “0,” the right end “1,000,” and no other marks. The numbers to be estimated (from Opfer and Siegler, 2007: 2, 5, 18, 34, 56, 78, 100, 122, 147, 150, 163, 179, 246, 366, 486, 606, 722, 725, 738, 754, 818, and 938) were centered above the midpoint of each line. After participants made each of their estimates by making a hatch mark on the number line, another problem was given.

##### Numerical recall

Participants listened to six short vignettes (see **Table 1**) and were asked to recall the numbers in the vignette after a brief distracter where children were asked to name colors, animals, fruits/vegetables, shapes, and modes of transportation presented on flash cards. Participants were told, “You will get to hear a short story. Try your best to remember all of the parts of the story because I will ask you some questions about the story later.” Each story involved three small (5, 18, 53, 79, 164, 237), medium (419, 487, 524, 548, 625, 632), or big numbers (725, 759, 817, 846, 938, 962). Numbers were presented randomly within vignettes, and each number was presented equally often with each vignette.

**Table 1:** Numerical recall vignettes.

Vignettes	Probe Questions
Beth wanted to find something to read at the public (her school’s) library. On a shelf at the public (school’s) library, she saw _____ magazines, _____ fiction books, and _____ non-fiction books.	How many magazines did Beth see?
	How many fiction books did Beth see?
	How many non-fiction books did Beth see?
Mr. Smith asked students in his school district (students at his school, second grade students at his school) how they liked to travel best. _____ liked airplanes, _____ like cars, and _____ liked trains.	How many students liked airplanes best?
	How many students liked cars best?
	How many students liked trains best?
Mrs. Conway asked students in her school district (students at her school, second grade students at her school) about their favorite foods. ____ students liked spaghetti best, _____ students liked pizza best, and _____ students liked chicken nuggets best.	How many students liked spaghetti best?
	How many students liked pizza best?
	How many students liked chicken nuggets best?
Some farmers (The farmer) planted different kinds of vegetables on (in) their farms (his farm, his garden). They (He) planted _____ carrots, _____ potatoes, and _____ cucumbers.	How many carrots did the farmers plant?
	How many potatoes did the farmers plant?
	How many cucumbers did the farmers plant?
Mr. Costa asked the students in his school district (students at his school, second grade students at his school) which card game they liked best. _____ liked Old Maid, _____ liked Go Fish, _____ liked Uno.	How many students liked Old Maid best?
	How many students liked Go Fish best?
	How many students liked Uno best?
Colleen washes the dishes at a restaurant. This month (week, weekend), she washed _____ forks, _____ cups, and _____ plates.	How many forks did Colleen wash?
	How many cups did Colleen wash?
	How many plates did Colleen wash?


#### Procedure

Children were tested individually during one 25-min experimental session occurring in a quiet room in their school; adults were tested individually during one 20-min experimental session in a laboratory on a college campus. Participants always completed the number line estimation task first, and no feedback was given on participants’ performance.

### Results and Discussion

#### Numerical Estimation

We first examined development of numerical estimation by measuring age-related changes in accuracy of number line estimates. Accuracy of estimates was indexed by percent absolute error (PAE), defined as: ([| to-be-estimated value – participant’s estimate|]/numerical range) ^∗^ 100. For instance, PAE = 45% if a child clicked at the location for 600 when asked to estimate the number 150 on a 0–1,000 number line, ([|150–600|]/1,000) ^∗^ 100. That is, the *higher* the PAE, the *less* accurate the estimates. As expected, accuracy of number line estimates improved substantially with age, *F*(2,102) = 80.87, *p* < 0.0001, η^2^ = 0.61 with Kindergartners’ PAE being 31% (*SD* = 9%), second graders’ 17% (*SD* = 8%), and adults’ 3% (*SD* = 0.9%).

Previous work (Siegler and Opfer, 2003) explained age-related changes in accuracy of number line estimates as stemming from a shift from logarithmic to linear mappings between symbolic and spatial values. To test this idea, we compared the fit of the logarithmic and linear regression functions for the relation between the median estimates of each age group and actual numeric value. Consistent with the logarithmic-to-linear shift hypothesis, we found that Kindergartners’ median estimates were best described by a logarithmic function (log *R*^2^ = 0.86, lin *R*^2^ = 0.47), second graders’ about equally by each function (log *R*^2^ = 0.884, lin *R*^2^ = 0.879), and adults by the linear function (log *R*^2^ = 0.66, lin *R*^2^ = 1.0).

To ensure that these fits did not arise from averaging over distinct cognitive profiles, we conducted an analysis to determine which participants approximately mapped numerical values to magnitudes (i.e., where the slope of the best-fitting linear function differed significantly from zero). If the slope of the best fitting linear function differed significantly from 0, participants were labeled “mappers.” If participants’ slope did not differ significantly from 0, they were labeled “non-mappers.” In the present study, 100% of adults, 98% of second graders, and 50% of Kindergartners were mappers. Likewise, 100% of adult mappers produced a series of estimates better fit by the linear than logarithmic function, and the same was true for 48% of second grade mappers, and 29% of Kindergarten mappers. The logarithmic function fit the data better than the linear function for 86% of Kindergarten non-mappers and for one second grade non-mapper. There were no adult non-mappers.

#### Numerical Recall

We next examined development of memory accuracy in numerical recall by measuring age-related changes in accuracy of memory. Accuracy of memory was again indexed by PAE, [(|to-be-remembered value – number participant remembered|)/1,000] ^∗^ 100. Please note that the *higher* the PAE the *less* accurate were the numbers recalled. Similar to number line estimation PAE, numerical recall PAE shows how far participants’ memories for numbers deviated from the numbers that were verbally presented in the short vignettes. As expected, accuracy of memory improved substantially with age, *r* = -0.63, *F*(1,102) = 67.51, *p* < 0.0001, with Kindergartners’ PAE being 35% (*SD* = 8%), second graders’ 19% (*SD* = 9%), and adults’ 7% (*SD* = 3%).

#### Relation Between Numerical Estimation and Memory Accuracy in Numerical Recall

Might improvements in memory accuracy—like improvements in accuracy of numerical estimates—be caused by a logarithmic-to-linear shift in representations of numerical value? Several observations suggest this might be the case.

**FIGURE 2:**
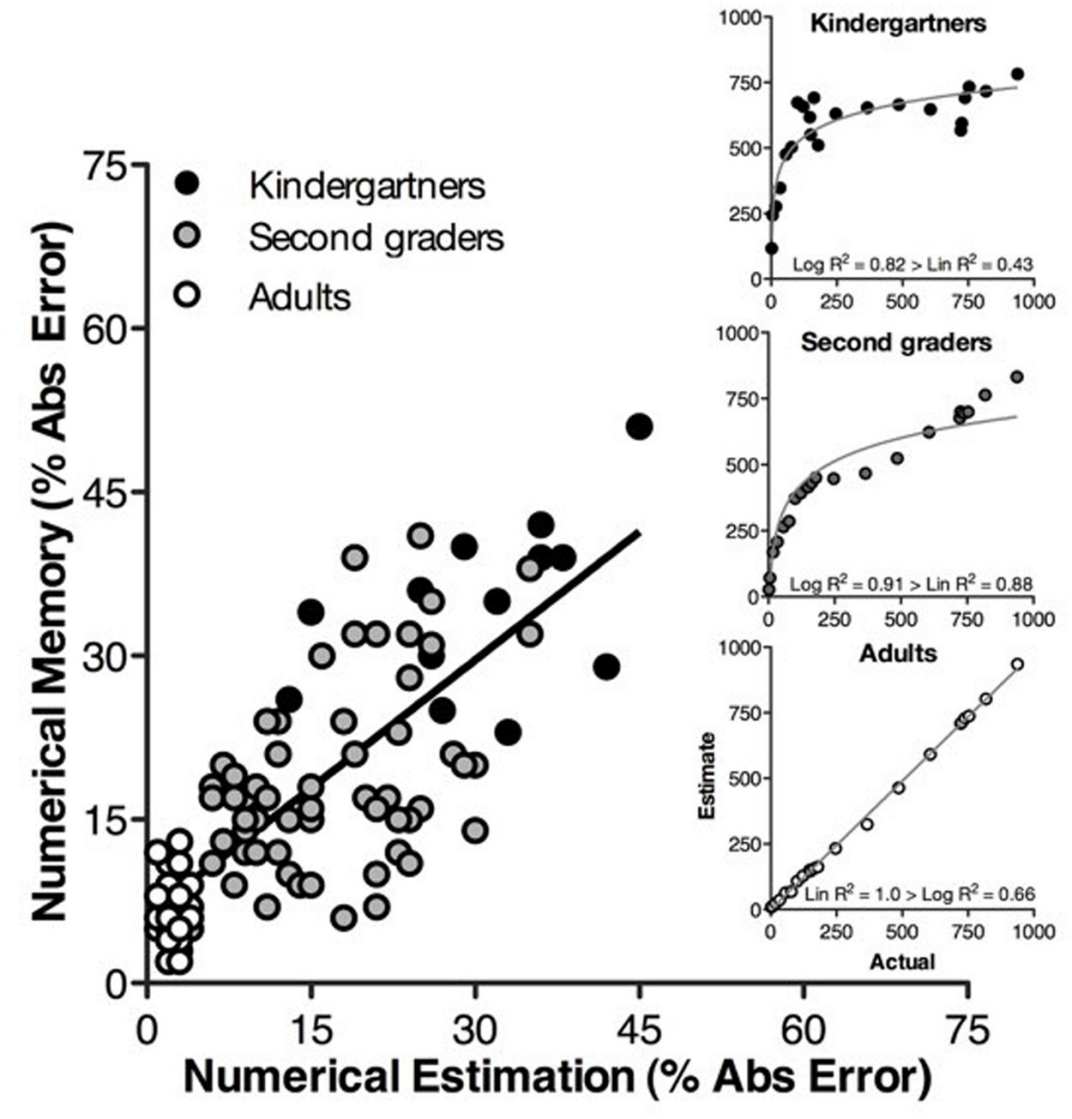
**Percent absolute error (PAE) on the numerical estimation task is strongly correlated with PAE on the numerical memory task for Kindergartners (black circles), second graders (gray circles), and adults (white circles).** The inset figures illustrate a logarithmic-to-linear shift in numerical estimation across the age range.

First, memory accuracy was highly correlated (*r* = 0.781) with performance in numerical estimation (**Figure 2**). Even when adults were removed from the analysis, number line estimation PAE and memory recall PAE were strongly correlated (*r* = 0.654). After statistically controlling for the effects of mapper status (e.g., Did participants approximately map numerical values to magnitudes on their placement of numbers on number lines?) and age, accuracy on number line estimation also positively predicted memory accuracy (slope = 0.66, *t* = 7.07, *p* < 0.0001).

A second set of observations came from the predicted effects of numerical magnitude on memory accuracy. That is, if numeric symbols are mapped with a constant noisiness to a logarithmically scaled mental number line, then signal overlap increases dramatically with numerical value, thereby leading to significant interference from adjacent values as the target number increases. In contrast, if numeric symbols are mapped with constant noisiness on a linearly scaled mental number line, then signal overlap is greatest for neighboring values but does not otherwise increase with numeric value. On a 0–1,000 mental number line, for example, the difference between the two representations would be greatest around 150 (see **Figure 1**), leading to a distinct pattern of predicted errors: for numbers greater than 150, use of a logarithmic representation would interfere much more with memory than use of a linear representation, whereas for numbers less than 150, accuracy would favor the logarithmic representation or neither representation (depending on overall noisiness of the mapping).

To test this prediction, we conducted a 2 (numerical range: below 150, above 150) × 2 (best fitting function on number line estimation: logarithmic, linear) ANOVA on PAE scores for recall. There was a main effect of numerical range, *F*(1,103) = 155.63, *p* < 0.0001, η^2^ = 0.52, and best fitting function, *F*(1,103) = 38.20, *p* < 0.0001, η^2^ = 0.27. The main effect for numerical range showed that children were more accurate on smaller as compared to bigger numbers (PAE = 6% vs. 21%). The main effect of best fitting function showed that children’s PAE was lower when they were best fit by the linear function (PAE = 9%) as compared to when they were best fit by the logarithmic function (PAE = 19%). There was also a significant numerical range × best fitting function interaction, *F*(1,103) = 41.97, *p* < 0.0001, η^2^ = 0.14. For numbers below 150, memory accuracy was high regardless of the numerical representation employed on the number line estimation task, *F*(1,103) < 1, *p* > 0.05. However, for numbers greater than 150, memory accuracy was much lower among participants who produced a logarithmic series of estimates on the number line estimation task than among participants who produced a linear series of estimates (PAE = 31% vs. 13%, respectively, *F*(1,103) = 71.51, *p* < 0.0001, η^2^ = 0.41). Thus, memory accuracy—particularly memory for large numbers—was associated with use of linear representations.

In summary, the proportion of children producing more logarithmic than linear estimation patterns declined with age. Results indicated that (1) children who produced a linear series of estimates were more accurate than children who produced a logarithmic series of estimates, (2) older children and adults were more likely to produce a linear series of estimates, (3) there was a strong correlation between numerical estimation performance and memory accuracy on a numerical recall task, and (4) developmental changes in numerical memory accuracy occurred much more for numbers greater than 150 than less than 150.

Findings from Thompson and Siegler (2010) and results from Study 1 provide converging correlational evidence that linear spatial-numeric associations are related to more accurate numerical recall. Additionally, the results show that age alone cannot account for the association between quality of representation and numerical memory, an issue that could not be explored in Thompson and Siegler’s data. This is important because it raises the possibility that manipulating the quality of numeric representations could improve numeric memory.

## Study 2: Effects of Training on Numerical Estimation and Memory Accuracy

### Method

#### Participants

Children from Study 1 who produced a logarithmic series of estimates on the number-line estimation task were included in Study 2 as were additional Kindergartners and second graders who were recruited to participate in the training procedure. Participants were 23 Kindergartners (*Mean age* = 6.23 years, *SD* = 0.39 years; 61% girls; 100% Caucasian; 48% were later assigned to the treatment group) and 64 second graders (*Mean age* = 8.31 years, *SD* = 0.34 years; 59% girls; 94% Caucasian, 3.1% Biracial, 1.6% Asian, 1.6% Hispanic; 47% were later assigned to the treatment group). The children were recruited from the same schools as in Study 1. This study was carried out in accordance with the recommendations of the Carnegie Mellon University IRB with written informed consent from the parents/guardians of all subjects and verbal assent from all child participants.

#### Tasks

The numerical estimation and recall tasks were equivalent to the tasks described in Study 1.

#### Procedure

Children were randomly assigned to a treatment group, who received corrective feedback on their placement of seven numbers (147, 150, 156, 163, 172, 179, and 187) on the number line, or a control group, who completed the same problems but without feedback on their estimates (see Opfer and Siegler, 2007, for a detailed description of the training procedure). During training, children made a hatch mark for the to-be-estimated number, and then the experimenter told the child whether the estimate was near (within 10%) or far (beyond 10%) from the correct location. After the experimenter indicated the correct placement and labeled the number the child mistakenly indicated, the child described why the corrected mark showed the right location for the number. After this training, both groups completed a 22-problem number-line posttest, followed by the numerical recall task described in Study 1.

### Results and Discussion

#### Effect of Feedback on Numerical Estimation

To assess the effectiveness of training, we conducted a 2 (test phase: pretest, posttest) × 2 (condition: control, treatment) × 2 (grade: Kindergarten, second grade) ANOVA on number line PAE scores. As expected, accuracy increased significantly from pretest to posttest, *F*(1,83) = 41.69, *p* < 0.0001, η^2^ = 0.30, with accuracy also being greater in the treatment than control condition, *F*(1,83) = 7.08, *p* < 0.01, η^2^ = 0.08, and greater for older than younger children, *F*(1,83) = 71.88, *p* < 0.0001, η^2^ = 0.46. Against the idea that pretest to posttest gains occurred through regression to the mean, we also observed a significant test phase × condition interaction, *F*(1,83) = 6.30, *p* < 0.05, η^2^ = 0.05. *Post hoc* analysis indicated that these gains from pretest to posttest were larger in the treatment group (*M* = 8%, *SD* = 7%) than in the control group (*M* = 3%, *SD* = 6%), *F*(1,85) = 16.31, *p* < 0.0001, η^2^ = 0.16. Finally, a test phase × condition × grade interaction, *F*(1,83) = 6.32, *p* < 0.05, η^2^ = 0.05, indicated that feedback reliably induced pretest-to-posttest gains among second graders (treatment: pretest, *M* = 21%, posttest, *M* = 11%; control: pretest, *M* = 22%, posttest, *M* = 20%) but not Kindergartners (treatment: pretest, *M* = 31%, posttest, *M* = 27%; control: pretest, *M* = 34%, posttest, *M* = 30%).

#### Transfer of Learning to Memory Accuracy in Numerical Recall

**FIGURE 3:**
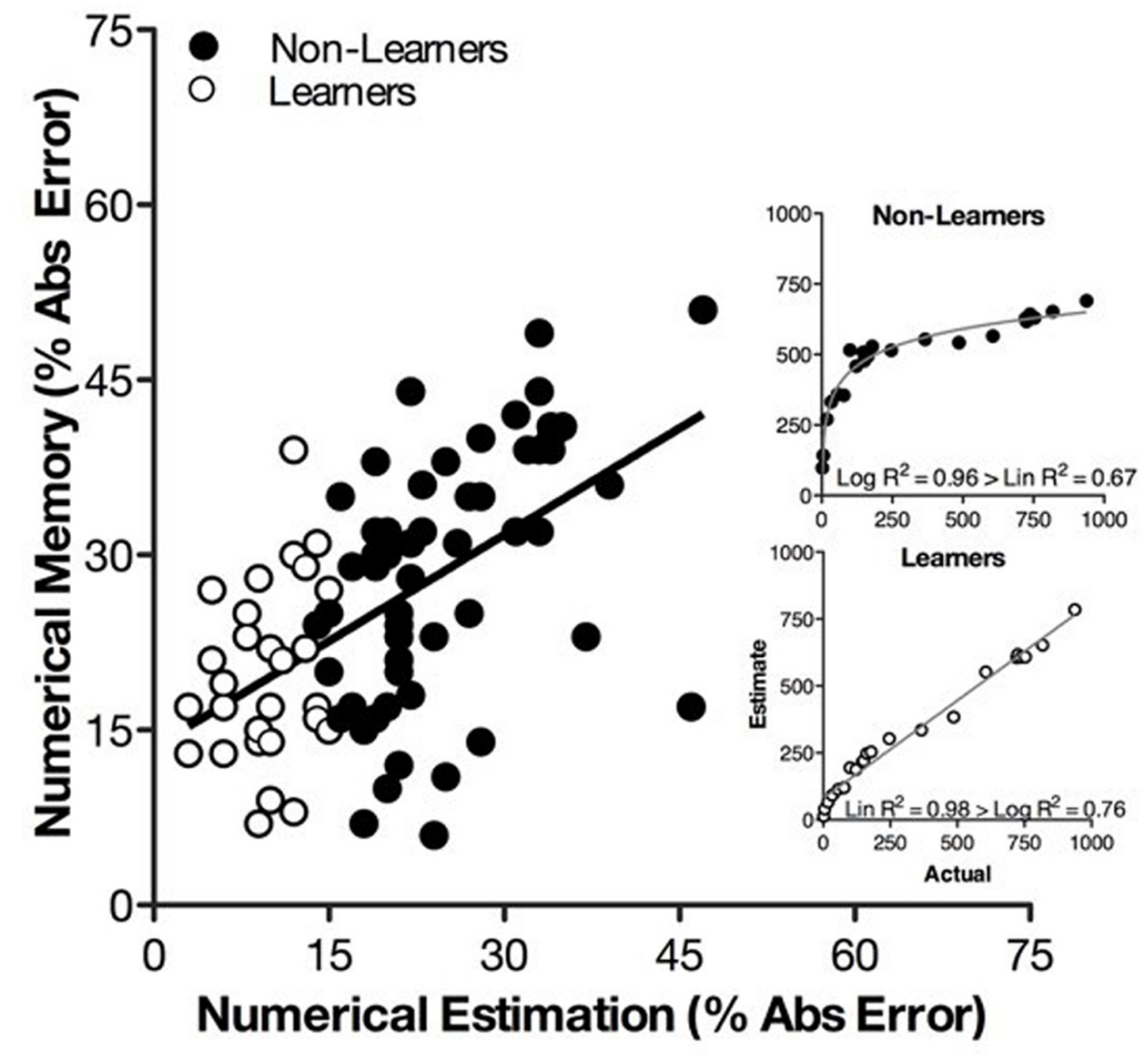
**Percent absolute error on the numerical estimation task is strongly correlated with PAE on the numerical memory task for non-learners (black circles) and learners (white circles).** The inset figures illustrate a logarithmic-to-linear shift in numerical estimation across non-learners and learners.

We next examined whether the logarithmic-to-linear shift that we induced in numerical estimation would also improve memory accuracy in a numerical recall task. As in Study 1, we found accuracy of numerical estimation and numerical recall were highly correlated (**Figure 3**), but we were interested in whether a causal connection existed. To examine this issue, we separated Kindergartners and second graders into two groups—learners (*N* = 31), those children who learned to produce a linear series of estimates on the number-line posttest, and non-learners (*N* = 56), those children who continued to produce a logarithmic series of estimates on the number-line posttest.

**FIGURE 4:**
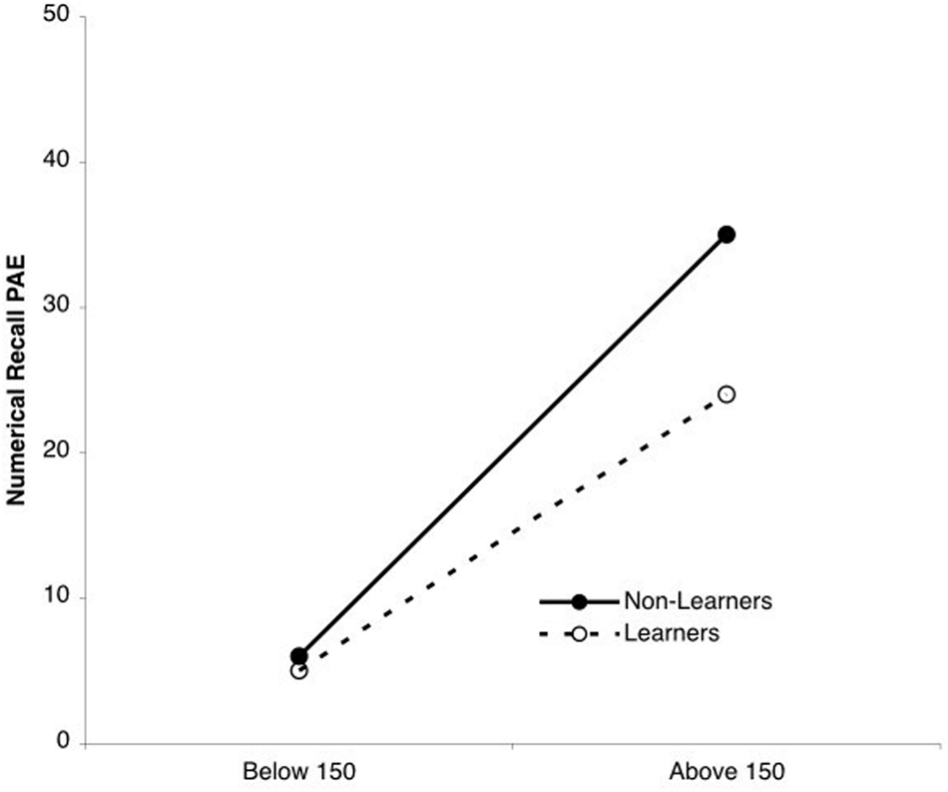
**Numerical range × learner status interaction.** Regardless of whether children had learned to produce a linear series of estimates on the number line estimation task, smaller numbers were remembered better than were larger ones. However, children who learned to produce a linear series of estimates remembered bigger numbers better than those children who did not learn to produce a linear series of estimates.

Our hypothesis was that the accuracy of learners’ recall would be higher than that of non-learners, and this difference would be especially strong for large numbers. To test this hypothesis, we conducted a 2 (numerical range: below 150, above 150) × 2 (learner status: non-learner, learner) ANOVA on PAE memory scores. As expected, memory was more accurate for small than large numbers, *F*(1,85) = 188.01, *p* < 0.0001, η^2^ = 0.67, and more accurate among learners than non-learners, *F*(1,85) = 12.81, *p* = 0.001, η^2^ = 0.13. Additionally, we observed a significant numerical range × learner status interaction (see **Figure 4**), *F*(1,85) = 7.94, *p* < 0.01, η^2^ = 0.03. For numbers below 150, memory accuracy on the numerical recall task was accurate regardless of whether children learned to produce a linear series of estimates on the number line estimation task (non-learners PAE = 6%, learners PAE = 5%), *F*(1,85) < 1, *p* > 0.05. For numbers greater than 150, however, non-learners were less accurate on the numerical recall task than learners (PAE = 35% vs. 24%, respectively, *F*(1,85) = 14.02, *p* < 0.0001, η^2^ = 0.14). Thus, as in Study 1, memory accuracy—particularly memory for large numbers—was associated with acquisition of linear representations.

Because there was a small effect size showing that learners had more accurate memory recall than non-learners, we investigated whether something other than learning linear representations could be responsible for this difference. We tested two alternative explanations. The first idea was that age alone improved recall. This idea seemed plausible because learners (*M* = 8.23, *SD* = 0.59) tended to be older than non-learners (*M* = 7.5, *SD* = 1.07), *t*(85) = 3.49, *p* < 0.001, *d* = 0.84, possibly leading them to have better memory. To test this idea, we examined second graders alone because roughly half of the 64 second graders (*n* = 29) qualified as learners, and their ages were very close (learners, *M* = 8.35, *SD* = 0.35; non-learners, *M* = 8.28, *SD* = 0.33, *t*(62) = 0.84, *p* > 0.05, *ns*). Here too we found that numerical recall was more accurate for learners than non-learners (learners, PAE = 19%, *SD* = 7%; non-learners, PAE = 24%, *SD* = 9%, *t*(62) = 2.01, *p* < 0.05, *d* = 0.62).

Another alternative explanation for why learners might have more accurately recalled numbers than non-learners was that feedback alone improved memory, regardless of whether it actually led to learning linear representations. Against this hypothesis, however, we found no main effect of feedback on memory accuracy (treatment, PAE = 18%, *SD* = 9%; control, PAE = 19%, *SD* = 12%; *F* < 1). Thus, actually learning linear representations from the feedback appeared both necessary and sufficient for the average child to improve memory accuracy.

## General Discussion

Previous work has indicated that a logarithmic-to-linear shift in children’s representations of symbolic quantities profoundly expands children’s quantitative thinking (for review, see Opfer and Siegler, 2012; for a discussion of alternative models, see Opfer et al., 2011, and Young and Opfer, 2011). It improves children’s ability to estimate the positions of numbers on number lines (Siegler and Opfer, 2003; Siegler and Booth, 2004; Berteletti et al., 2010; Thompson and Opfer, 2010), to estimate the measurements of continuous and discrete quantities (Opfer et al., 2010; Thompson and Siegler, 2010), to categorize numbers according to size (Opfer and Thompson, 2008), and to estimate and learn the answers to arithmetic problems (Booth and Siegler, 2008). Recent work has also indicated that the logarithmic-to-linear shift is associated with improved memory for numbers (Thompson and Siegler, 2010), but it was unclear whether there was a causal link between the representations of numerical magnitude and memory for numbers.

We found evidence that a logarithmic-to-linear shift in estimating the position of numbers on number lines was both correlated with and likely causally related to improved memory for numbers. In Study 1, linearity of numerical estimates increased with age, and the more linear children’s magnitude representations were, the more closely their memory of the numbers approximated the numbers presented. These results provided a replication of earlier results, and they also revealed that performance on the number line estimation task mediated the relation between age and memory accuracy on a numerical recall task.

To test the idea that linear magnitude representations were causally related to number memory, we trained half of the children on a linear spatial-numeric association on the number line task. Consistent with there being a causal link between numerical magnitude representations and memory for numbers, children who learned to represent numbers as increasing linearly with numeric magnitude also improved their memory for numbers. This improvement was particularly large for numbers greater than 150, though children were not given feedback on their estimates in this range. Theoretically, this finding is interesting because it is a prediction that comes from the logarithmic-to-linear shift account (see **Figure 1**). That is, the linear representation makes it easier to discriminate numbers at the high end of the range, whereas the logarithmic representation compresses numbers at the high end of the range. This result is important because it provides a specific mechanism for developmental change in the accuracy of memory for numbers (i.e., improved knowledge of numerical magnitudes).

One limitation of our study is that we do not know how our participants performed on a standardized measure of mathematics achievement because the state of Pennsylvania does not mandate mathematics achievement tests for children in this age range. It is possible that those children who were more likely to learn a linear representation also possessed higher overall mathematics achievement, and this can be investigated in future research. Further, we do not know how broad and durable the improvements in memory accuracy are after children receive training on the number line estimation task. We improved memory for the predicted range of numbers, but we do not know if other numeric ranges might also be impacted by our training procedure. In fact, to our knowledge, the numerical cognition literature does not include any published data on the durability of number line estimation training in general because most training studies occur within one experimental session.

Beyond demonstrating that linear spatial-numeric associations improve memory for numbers, we believe the present results also help to explain the positive relation between linear numeric magnitude representations and arithmetic proficiency (Booth and Siegler, 2008; Schneider et al., 2009; Siegler and Ramani, 2009). That is, if learning linear spatial-numeric associations improves memory for numbers in vignettes, it is highly likely it also improves memory for numbers in other contexts, such as memorizing arithmetic facts, as well as memory for fractions, where development is more protracted (Opfer and DeVries, 2008; Thompson and Opfer, 2008; Siegler et al., 2011). Thus, the present results suggest a plausible explanation for the observed association between numerical estimation and mathematics achievement, though this remains an important issue for future research.

## Author Contributions

CT and JO designed the study, CT collected the data, CT and JO analyzed the data, CT and JO wrote the manuscript.

## Conflict of Interest Statement

The authors declare that the research was conducted in the absence of any commercial or financial relationships that could be construed as a potential conflict of interest.
